# Retraction notice to “Rapid cutaneous remission response to tofacitinib and systemic corticosteroids in VEXAS syndrome with a novel UBA1 mutation” [JAAD Case Reports 68 (2026) 105-107]

**DOI:** 10.1016/j.jdcr.2026.05.019

**Published:** 2026-05-12

Zehai Wang,^a^ Hui Shi,^b^ Yanyun Jiang^a^

^a^Department of Dermatology, Sun Yat-sen Memorial Hospital, Sun Yat-sen University, Guangzhou, China; ^b^Department of Rheumatology and Immunology, Ruijin Hospital, School of Medicine, Shanghai Jiao Tong University Shanghai, China

This article has been retracted: please see Elsevier Policy on Article Withdrawal (https://www.elsevier.com/about/policies/article-withdrawal).

This article has been retracted at the request of the **authors**.

Following the receipt of a Letter to the Editor and subsequent internal review, the authors have re-evaluated the genetic data presented in this report. The UBA1 variant described in the manuscript was identified solely by next-generation sequencing of peripheral blood, with a variant allele frequency of 100%, and without additional deep sequencing, tissue-based validation, or lineage-specific analysis. Based on currently available evidence, the authors recognize that the somatic status and pathogenic relevance of this variant cannot be reliably established. As the interpretation of this genetic finding represents a central component of the article, the authors have requested retraction to maintain high standards of scientific accuracy and integrity.

The request was supported by the Editor-in-Chief after confirming with all authors their wish to retract.

